# Herpes simplex virus infection induces necroptosis of neurons and astrocytes in human fetal organotypic brain slice cultures

**DOI:** 10.1186/s12974-024-03027-5

**Published:** 2024-02-01

**Authors:** Ahmad S. Rashidi, Diana N. Tran, Caithlin R. Peelen, Michiel van Gent, Werner J. D. Ouwendijk, Georges M. G. M. Verjans

**Affiliations:** HerpesLabNL of the Department of Viroscience (Room Ee1720a), Erasmus Medical Center, Dr. Molewaterplein 40, 3015 GD Rotterdam, The Netherlands

**Keywords:** Herpes simplex virus, Encephalitis, Human brain, Organotypic cultures, Neurotropism, Neurovirulence, Programmed cell death, Necroptosis

## Abstract

**Background:**

Herpes simplex virus (HSV) encephalitis (HSE) is a serious and potentially life-threatening disease, affecting both adults and newborns. Progress in understanding the virus and host factors involved in neonatal HSE has been hampered by the limitations of current brain models that do not fully recapitulate the tissue structure and cell composition of the developing human brain in health and disease. Here, we developed a human fetal organotypic brain slice culture (hfOBSC) model and determined its value in mimicking the HSE neuropathology in vitro.

**Methods:**

Cell viability and tissues integrity were determined by lactate dehydrogenase release in supernatant and immunohistological (IHC) analyses. Brain slices were infected with green fluorescent protein (GFP-) expressing HSV-1 and HSV-2. Virus replication and spread were determined by confocal microscopy, PCR and virus culture. Expression of pro-inflammatory cytokines and chemokines were detected by PCR. Cell tropism and HSV-induced neuropathology were determined by IHC analysis. Finally, the in situ data of HSV-infected hfOBSC were compared to the neuropathology detected in human HSE brain sections.

**Results:**

Slicing and serum-free culture conditions were optimized to maintain the viability and tissue architecture of ex vivo human fetal brain slices for at least 14 days at 37 °C in a CO_2_ incubator. The hfOBSC supported productive HSV-1 and HSV-2 infection, involving predominantly infection of neurons and astrocytes, leading to expression of pro-inflammatory cytokines and chemokines. Both viruses induced programmed cell death—especially necroptosis—in infected brain slices at later time points after infection. The virus spread, cell tropism and role of programmed cell death in HSV-induced cell death resembled the neuropathology of HSE.

**Conclusions:**

We developed a novel human brain culture model in which the viability of the major brain-resident cells—including neurons, microglia, astrocytes and oligodendrocytes—and the tissue architecture is maintained for at least 2 weeks in vitro under serum-free culture conditions. The close resemblance of cell tropism, spread and neurovirulence of HSV-1 and HSV-2 in the hfOBSC model with the neuropathological features of human HSE cases underscores its potential to detail the pathophysiology of other neurotropic viruses and as preclinical model to test novel therapeutic interventions.

**Supplementary Information:**

The online version contains supplementary material available at 10.1186/s12974-024-03027-5.

## Introduction

Various pathogens grouped under the acronym ‘TORCH’ [*Toxoplasma gondii*, Others (e.g. parvovirus B and Zikavirus and Rubella virus), Cytomegalovirus and Herpes simplex viruses (HSV)], remain a substantial threat to newborns and fetuses worldwide [1]. Besides rubella virus, the neurotropic HSV are the second most prevalent TORCH pathogens during pregnancy that can cause neonatal herpes associated with serious morbidity (e.g. neurological and disseminated disease) and mortality [2]. The global incidence rate of neonatal HSV infection is about 10 cases per 100,000 livebirths and 0.82 deaths per 1,000,000 births in industrialized countries due to either HSV-1 or HSV-2 [2, 3]. While the majority of neonatal HSV infections are acquired at birth, congenital HSV due to intrauterine HSV infection can occur leading to severe abnormalities affecting multiple organs including the brain [4, 5].

Whereas progress have been made in the early diagnosis and antiviral treatment of neonatal HSE, still about 60% of patients experience lifelong neurological sequela including cognitive impairment and epilepsy [2–4]. Thus, besides development of better prevention strategies, including effective HSV vaccines and prevention of maternal infection during pregnancy, there is an unmet need to identify the detrimental virus and host parameters involved in the pathogenesis of neonatal HSE as potential targets of therapeutic intervention [3, 6]. Various in vitro and in vivo models have been developed to address this issue, but most if not all fail to mimic HSV neurovirulence in the developing human brain [7–9]. Human 2D brain cell models lack the cell complexity of brain tissue, experimental rodent models are evidently lacking the human component and the more recently used human brain organoid models lack microglia and vasculature [7, 10–12]. Organotypic brain slice cultures (OBSC), which largely fulfill the shortcomings of the aforementioned models, have been successfully applied for rodents to both unravel the pathogenesis of various neurological diseases as well as provide the unique opportunity to determine the efficacy and safety of various therapeutic interventions in parallel using a limited number of animals [13–15]. In comparison, studies on human OBSC are limited not only by the scarce availability of clinical brain specimens, but also due to technical drawbacks including limited cell viability and aberrant tissue structure caused by long-term culture in the presence of serum in culture media [7, 12, 16]. To overcome these limitations, the use of serum-free medium combined with various growth factors is preferred to create a more optimal and reproducible environment while minimizing glial reactivity [17–19].

The aim of the current study was twofold. First, to develop a novel human fetal OBSC (hfOBSC) model that recapitulates the complexity of the developing human brain and that can be successfully cultured in serum-free medium for couple of weeks in vitro. Second, to study the cell tropism, virus spread and neurovirulence of HSV-1 and HSV-2 in hfOBSC and to compare with archived brain specimens of neonatal HSV cases by in situ analysis.

## Materials and methods

### Archived human brain specimens

Formalin-fixed and paraffin-embedded (FFPE) brain samples were obtained for diagnostic purposes from two fatal HSE patients, including one adult HSV-1- and one neonatal HSV-2-associated HSE case. The brain samples were provided by the BioBank of the Amsterdam University Medical Center (Amsterdam, The Netherlands). According to the institutional “Opt‐Out” system, which is defined by the National “Code of Good Conduct” [Dutch: Code Goed Gebruik, May 2011], these surplus human brain tissues were made available for the current study.

### Human fetal organotypic brain slice cultures

Human fetal brain tissue from legally terminated second trimester pregnancies (17–20 weeks) was obtained by the HIS-Mouse Facility of Academic Medical Center (AMC; Amsterdam, The Netherlands), after written informed consent of the mother for the tissue’s use in research and with approval of the Medical Ethical Review Board of the AMC (MEC: 03/038) and Erasmus MC (MEC-2017-009). Study procedures were performed according to the Declaration of Helsinki, and in compliance with relevant Dutch laws and institutional guidelines. The tissues obtained were anonymized and non-traceable to the donor. On request by the researchers, only gender and gestational age are provided. Abortions were not performed for medical indications and fetuses did not have any major anatomical deformities (checked by ultrasound prior to the procedure) or trisomy. The procedure was performed by in utero dissection and removal of fetal tissue specimens did not enable recovery of intact organs. Generation of brain slices was performed largely as described previously [20], with some modifications as explained below and illustrated in Additional file 1: Fig. S1. Prior to selecting cortical brain tissue, exterior blood vessels and meninges were removed. The tissue was then placed between agarose blocks to mechanically support the tissue fragments during the cutting process. Brain tissue fragments (approximately 0.5 × 0.5 cm in size) were cut into 350-µm-thick slices using a vibratome (Leica; type VT1200S) in artificial cerebrospinal fluid (aCSF) under constant oxygenation (95% O_2_, 5% CO_2_) as described previously [20]. Brain slices were transferred to 12-mm Transwells with polyester membrane inserts (0.4 µm pore size; Corning) and recuperated for 1 h in recovery medium that was composed of a 7:3 (v/v) mixture of Neurobasal media and advanced DMEM/F12 culturing medium (both Life Technologies) supplemented with 20% heat-inactivated fetal bovine serum (FBS; Sigma, cat: F7524, lot: 0001644044) and antibiotics [15]. Previous studies have shown that inclusion of a short recovery phase in serum-containing medium is critical for reestablishing brain tissue homeostasis and long-term culture viability [14, 21, 22]. After 1-h incubation in a CO_2_ incubator at 37 °C, the recovery medium was replaced with optimized hfOBSC serum-free culture medium consisting of a 7:3 (v/v) mixture of Neurobasal media and advanced DMEM/F12 culturing medium, B27 (2% v/v), N2 (1% v/v), glutaMAX 100x (1% v/v, all from Life Technologies), primocin (1:500, Invitrogen), TGF-β2 human (2 ng/mL, ProspecBio), cholesterol (1.5 µg/mL, Sigma Aldrich), human recombinant m-CSF (100 ng/mL, Peprotech), BDNF (50 µg/mL), NT-3 (10 ng/mL, Peprotech), FGF2 (10 ng/mL, R&D Systems) and EGF (10 ng/mL, R&D Systems). In the Transwell system, 750 µl was added to the basolateral compartment and 50 µl added to the apical compartment to counteract the vaporization nutrition in the tissue under incubation. The culture medium of both the basolateral and the apical compartment of the hfOBSC was refreshed every 48 h.

### Brain cell viability assays

The viability of the hfOBSC was determined by lactate dehydrogenase (LDH) and 3-(4,5-dimethylthiazol-2-yl)-2,5-diphenyltetrazolium bromide (MTT) assays. The LDH assays were performed on conditioned medium of hfOBSC cultures using the CyQUANT™ LDH Cytotoxicity Assay kit (Invitrogen) according to the manufacturer’s protocol. LDH levels of brain slices incubated in lysis buffer according to the manufacturer’s instructions were included as the positive control (CTRL) indicative of maximum cell death. To assess metabolic activity, brain slice cultures were treated with MTT (0.5 g/L, Abcam) and incubated for 45 min at 37 °C before imaging using Zeiss LSM 700 confocal microscope (Zeiss).

### HSV infection and IFN-γ treatment of human fetal organotypic brain slice cultures

Recombinant HSV-1 strain 17 expressing green fluorescent protein (GFP) N-terminally tagged to VP16 (HSV-1-GFP), and HSV-2 (strain 333 variant ZAG) that contains a GFP expression cassette under the control of a human cytomegalovirus promoter inserted in an intergenic region between *UL3* and *UL4*, were propagated on human retinal pigment epithelial (ARPE-19) cells to obtain cell-free virus stocks, as previously described [23, 24]. The hfOBSC were infected with 100 plaque-forming units (PFU)/ml or 10^6^ PFU/ml of cell-free HSV-1-GFP or HSV-2-GFP. After 1-h incubation at 37 °C, the inoculum was removed, and the brain slices were washed with PBS and subsequently maintained in culture medium for 72 to 96 h post-infection (hpi) at 37 °C in a CO_2_ incubator. Infection was monitored by collecting supernatants from both apical and basolateral side every 24 h for quantitative real-time DNA PCR (qPCR) and assessing GFP expression using a fluorescent microscope (Zeiss AXIO inverted microscope). In some experiments, brain slices were treated with 1,000 U/ml of recombinant human IFN-γ (Peprotech, 300-02) for 48 h as positive control to induce microglia activation [25]. At the indicated time points, the hfOBSC were fixed in PBS containing 4% paraformaldehyde and embedded in paraffin for histological analyses.

### Quantitative real-time PCR for HSV DNA and host transcripts detection

The viral loads of HSV-1 and HSV-2 DNA in supernatants and gene expression of cytokine and chemokines in HSV-infected brain slices were quantified by Taqman quantitative real-time PCR (qPCR) as described previously [26–28]. In brief, for gene expression the total RNA was extracted from the HSV- and mock-infected brain slices using the Total RNA isolation kit (Omega Biotek) according to the manufacturer’s instructions. Reverse transcription using oligo dT and subsequent qRT-PCR were performed using the purified RNA and the SuperScript III Platinum One-Step qRT-PCR kit with ROX (Invitrogen) on a 7500 Fast Real-Time PCR Machine (Applied Biosystems). To detect human transcripts Taqman fast Advanced mastermix (Thermo Fisher scientific) was performed using human primers/probes specific for the following targets: *IL1B* (interleukin-1 β, IL-1β), *IFNB1* (IFN-β), *TNFA* (tumour necrosis factor alpha, TNF-α), *IL6* (interleukin-6, IL-6), *CXCL10* (CXCL10, IP-10), and *HLA-DRA* (human major histocompatibility class II, MHC-II) purchased from Integrated DNA Technologies (IDT).

HSV-1 and HSV-2 DNA loads in supernatants were quantified by Taqman quantitative real-time PCR (qPCR) as described previously [28]. In brief, assays were performed in Taqman fast Advanced mastermix (Thermo Fisher scientific) using primers/probes specific for the following targets: HSV-1 gene *US4* (glycoprotein G) and HSV-2 gene *US6* (glycoprotein D) [28]. The HSV-specific qPCR was performed on a 7500 Real-time PCR system (Applied Biosystems) and the PCR cycling program used consisted of 5 min at 50 °C and 2 min at 95 °C, followed by 40 cycles of 3 s at 95 °C and 30 s at 60 °C.

### In situ analysis of brain slices and tissues

The FFPE brain slices and archived brain sections of two HSE cases were serially sectioned at 4 µm (Thermo Shandon Limited, HM 340 E) thickness. Heat-induced antigen retrieval was performed using citric acid buffer (pH 6.0). Consecutive tissue sections from three different levels of the brain slices (i.e. apical, middle and basal level) were immunohistochemically stained (IHC) with the following primary antibodies: polyclonal rabbit anti-Iba1 (microglia marker; Wako, 1:500), anti-GFAP (glial fibrillary acidic protein, astrocyte marker; Dako, 1:500), monoclonal rabbit anti-Olig2 (oligodendrocyte transcription factor 2, oligodendrocyte marker; clone EPR2673, Abcam, 1:200) or polyclonal guinea pig anti-MAP2 (microtubule-associated protein 2, neuron marker; Synaptic Systems, 1:300), major histocompatibility class II protein (MHC-II) as prototypic activation marker monoclonal mouse anti-HLA-DP/DQ/DP (clone CR3/43; DAKO, 1:250). Next, sections were washed and incubated with the appropriate secondary antibody including biotinylated rabbit anti-mouse Ig (Dako, 1:200), biotinylated goat anti-rabbit Ig (Dako, 1:200), or horseradish peroxidase (HRP)-conjugated rabbit anti-guinea pig IgG (H + L) (Invitrogen, 1:200), respectively. HRP-labeled streptavidin (Dako, 1:300) was applied to sections with biotinylated antibodies. 3-amino-9-ethylcarbazole was used as a substrate. Sections were counterstained with hematoxylin, mounted with Kaiser’s glycerol, and scanned using the Hamamatsu NanoZoomer 2.0 HT (Hamamatsu).

Immunofluorescent (IF) stainings were performed on consecutive sections treated with TrueBlack® (Biotium) after antigen retrieval to decrease autofluorescence, according to the manufacturer’s protocol. The following primary antibodies were used for IF: polyclonal chicken anti-GFAP (Abcam, 1:500), polyclonal rabbit anti-Iba1 (Wako, 1:500), monoclonal rabbit anti-Olig2 (clone EPR2673, Abcam, 1:200), polyclonal guinea pig anti-MAP2 (Synaptic Systems, 1:300), and FITC polyclonal goat anti-GFP (Abcam, 1:200). The primary antibodies were labeled with the appropriate secondary antibodies from Invitrogen: Rabbit anti-GFP, AlexaFluor™ 647 (AF647, 1:250)- and AF555-conjugated goat anti-guinea pig (1:250) or chicken IgG or donkey anti-Rabbit IgG (1:250), respectively. Nuclei were stained with Hoechst 33342 Solution. Images were taken using a Leica Stellaris 5 Low Incidence Angle confocal microscope.

### Terminal deoxynucleotidyl transferase-mediated dUTP nick end labeling (TUNEL) assay

TUNEL assay was performed using the ApopTag® Plus in situ apoptosis fluorescein S7111 detection kit (Sigma) according to the manufacturer’s protocol, following instructions for a combined IF staining. Sections were treated with TrueBlack® (Biotium) after antigen retrieval to decrease autofluorescence, followed by staining with the following primary antibodies: polyclonal guinea pig anti-MAP2 (Synaptic Systems, 1:300), polyclonal chicken anti-GFAP (Abcam, 1:500), polyclonal rabbit anti-CC3 (cleaved caspase 3 protein; Cell Signalling Technology, 1:300), polyclonal rabbit anti-pMLKL (phosphorylation of mixed lineage kinase domain-like protein; Abcam, 1:250) and monoclonal mouse anti-GSDMD (gasdermin D protein; Abnova, 1:250). Subsequently, slides were incubated with the following secondary antibodies (Invitrogen): AF555-conjugated donkey anti-rabbit IgG (1:250), AF647-conjugated goat anti-guinea pig IgG (1:250), AF647-conjugated goat anti-chicken IgG (1:250) or AF647 goat anti-mouse IgG2a (1:250), respectively.

### Image processing

Following confocal imaging, the acquired images were subjected to quantitative analysis using ImageJ software (version 1.53t, National Institutes of Health, Bethesda, MD) and a customized script. Individual channels were analyzed after splitting the images. For Hoechst staining, a Gaussian blur filter of 1 was applied to remove noise, and a ‘Moments dark’ threshold was utilized for image segmentation. Particle quantification was performed exclusively on particles larger than 5 μm. TUNEL staining was processed by subtracting the median of 6 to eliminate background noise, followed by the application of a Gaussian blur of 1 and a ‘Yen dark’ threshold for accurate image segmentation. Quantification was limited to particles positive for both Hoechst and TUNEL staining. In the case of CC3, pMLKL, and GSDMD staining, background noise was efficiently removed by applying a median of 16 and a Gaussian blur of 0.5. A ‘Yen dark’ threshold was then utilized to identify particles larger than 20 μm. The percentage of positive cells was calculated by determining the ratio of positive cells to the total number of Hoechst-positive cells. Finally, all processed images were converted to mask, and the watershed method was employed to accurately separate particles for comprehensive analysis. Phenotypes of infected cells were identified and quantified using the classifications function on the QuPath 0.3.2 software [29].

### Statistical analysis

Data were analyzed using Prism 8.0.2 (GraphPad). A two-tailed Student’s *t* test was used to test for statistical significance as indicated in the figure legends. The *p* values were assigned: *p* > 0.05 (ns, not significant); *p ≤ 0.05; **p ≤ 0.01; ***p ≤ 0.001.

## Results

### Development of a human fetal organotypic brain slice culture platform

**Fig. 1:**
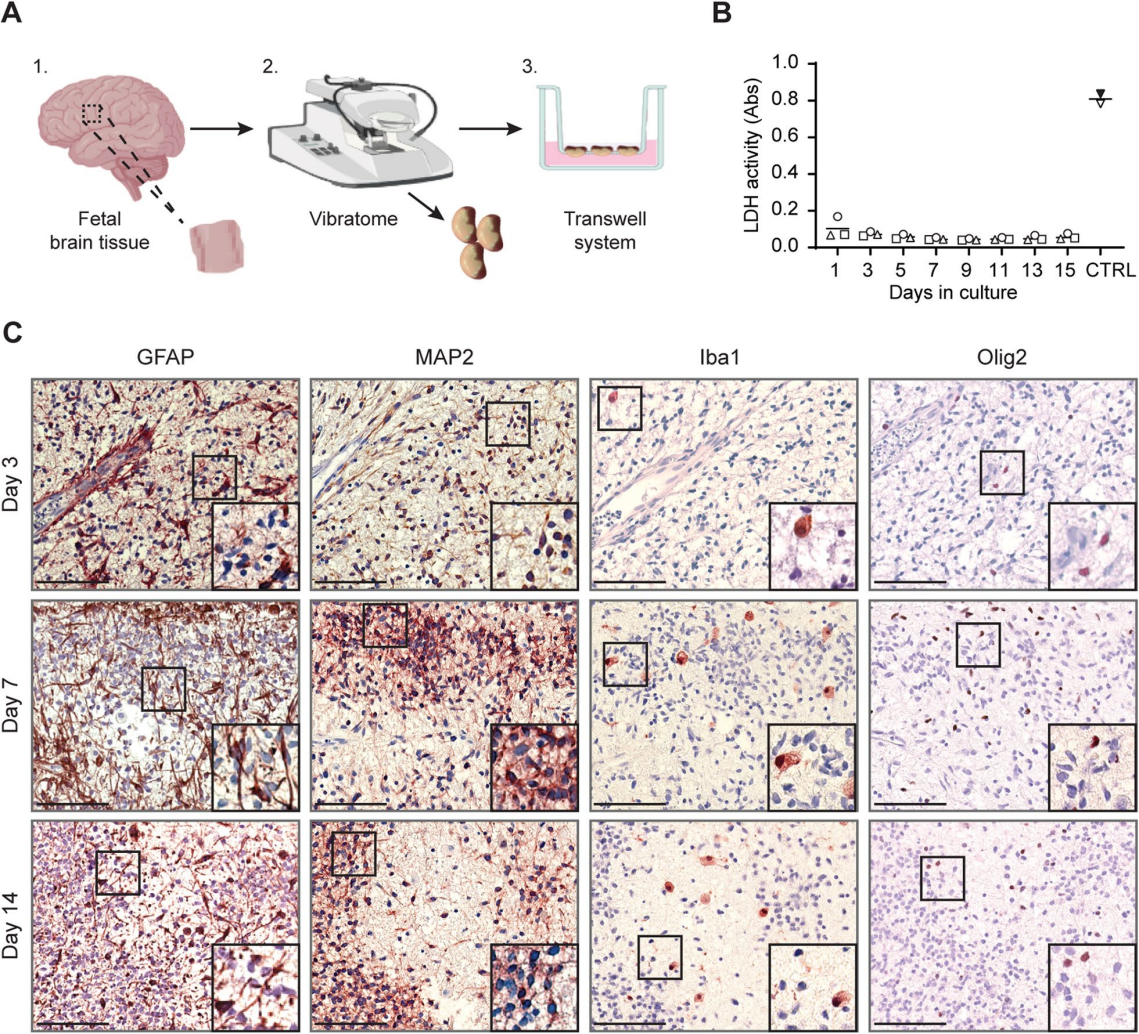
Human fetal organotypic brain slice cultures maintain brain tissue architecture and viability for at least 14 days in culture. **A** Schematic representation of the human fetal organotypic brain slice (hfOBSC) cultivation platform. Slices of 350-µm thick were cut from human fetal brain tissue (17–20 weeks of gestation) and cultured in a transwell system containing serum-free culture medium for over 14 days in a CO_2_ incubator at 37 °C. **B** Lactate dehydrogenase (LDH) assay showing limited cell death in human brain slices at the indicated days (1–14) of culture. Data are presented as mean absorbance ± SD of three independent subjects, including LDH levels of lysed slices as positive control (CTRL) indicative for maximum cell death. **C** Immunohistochemistry staining (brown color) on longitudinal sections of hfOBSC slices at days 3, 7, or 14 of culture using antibodies specific to astrocyte marker GFAP, neuron marker MAP2, microglia marker Iba1 or oligodendrocyte marker Olig2. Images are representative of two independent subjects and show stainings of consecutive longitudinal tissue sections obtained from three levels of the cultured hfOBSC (i.e. apical, middle and basal level of the brain slice). Scale bar: 100 µm

The aim of this study was to develop a human fetal OBSC (hfOBSC) platform that recapitulates the complexity of the developing human brain and that can be successfully cultured in serum-free medium for a couple of weeks. Hereto, human fetal brain tissue, obtained at 17 to 20 weeks of gestation, was cut with a vibratome into 350-µm-thick slices of 25 mm^2^ in size (Additional file 1: Fig. S1). Brain slices were recuperated in aCSF under constant oxygenation to reduce cell damage due to hypoxia [30, 31] (Fig. 1A). The slices were cultured in the upper compartment of a Transwell insert in a CO_2_ incubator at 37 °C, a well-established method to culture organotypic tissue slices [15, 32, 33]. Addition of a minimal amount (50 µl) of medium to this compartment allowed optimal oxygenation of the tissue, whereas the Transwell membrane ensured diffusion of nutrients from the large volume of media from the lower compartment into the tissue. To assess the overall viability of the hfOBSC, LDH concentrations measured in conditioned medium showed negligible cellular cytotoxicity throughout 14 days of culture (Fig. 1B). In line with this, the MTT assay, which measures cellular metabolic activity, showed no signs of reduction in cellular metabolism during 14 days of culture (Additional file 1: Fig. S1H).

Next, we determined whether these culture conditions preserved the four main CNS cell types (i.e., neurons, astrocytes, oligodendrocytes, and microglia) and overall brain tissue architecture in cultured hfOBSC. Immunohistochemical staining was performed on sections derived from three different levels (i.e., apical, middle and basal layer) of the hfOBSC to identify the presence and spatial orientation of GFAP^+^ astrocytes, MAP2^+^ neurons, Iba1^+^ microglia and Olig2^+^ oligodendrocytes. All four major cell types were detected throughout the hfOBSC and their morphology did not change during 14 days of culture. Consistent with existing literature, we observed similar numbers of neurons and glia cells, which were predominantly composed of astrocytes followed by lower numbers of oligodendrocytes and microglia [34, 35]. The microglia population contained both ramified and amoeboid-like Iba1^+^ cells, but no astrogliosis or microgliosis was observed at later times (Fig. 1C). In conclusion, the slicing and serum-free culture conditions developed maintained the viability and overall integrity of ex vivo human fetal brain slices for at least 14 days in culture.

### HSV-1 and HSV-2 predominantly infect neurons and astrocytes in hfOBSC

**Fig. 2:**
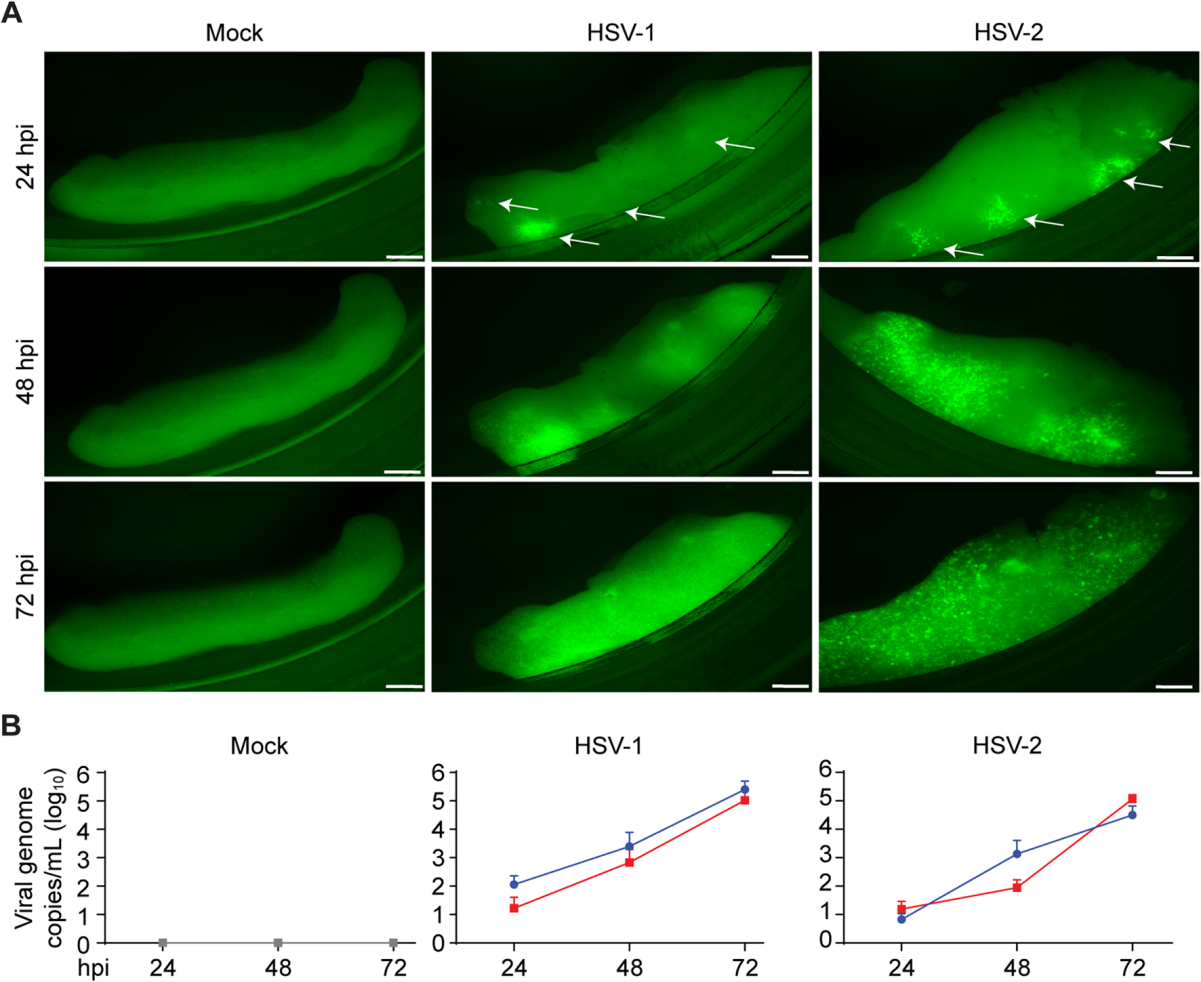
Human fetal organotypic brain slices cultures support productive HSV-1 and HSV-2 infection. **A** Microscopy analysis of green fluorescent protein (GFP) expression as a marker of cells infected with GFP-expressing HSV-1 or HSV-2. White arrow heads indicate foci of initial infection. Images shown are fluorescent microscopy pictures of the same human fetal organotypic brain slice (hfOBSC) at 24, 48 and 72 h post-infection (hpi) and are representative of hfOBSC cultures of five subjects. Scale bar: 500 µm. **B** Quantification of HSV-1 and HSV-2 DNA loads in culture medium was quantified by Taqman quantitative real-time PCR (qPCR) as described previously [27] of representative hfOBSC cultures. Data are presented as mean viral DNA copies/mL ± SD of two independent subjects (blue and red color)

The second aim of our study was to determine the cell tropism, virus spread and neurovirulence of HSV-1 and HSV-2 in hfOBSC and to compare these with archived brain specimens of human HSE cases by in situ analysis. Recombinant HSV strains encoding GFP were used to determine whether the cultured brain slices were permissive to productive HSV-1 and HSV-2 infection. The use of GFP reporter viruses facilitated both the identification of infected cells and to monitor virus spread longitudinally by fluorescence microscopy. The introduction of the GFP cassette does not affect viral replication in vitro [23, 24]. Infections were initiated at day 3 of culture (DIV3) to allow the brain slices to equilibrate following the slicing procedure. Following inoculation of the brain slices with 100 PFU/mL HSV-1-GFP or HSV-2-GFP, several foci of infected GFP-expressing cells were observed at 24 hpi, from which infection spread throughout the tissue until 72 hpi (Fig. 2A). Quantitative PCR analysis showed an exponential increase in viral DNA in the supernatant of hfOBSC over time, which demonstrated productive HSV-1 and HSV-2 infection of hfOBSC (Fig. 2B).

**Fig. 3:**
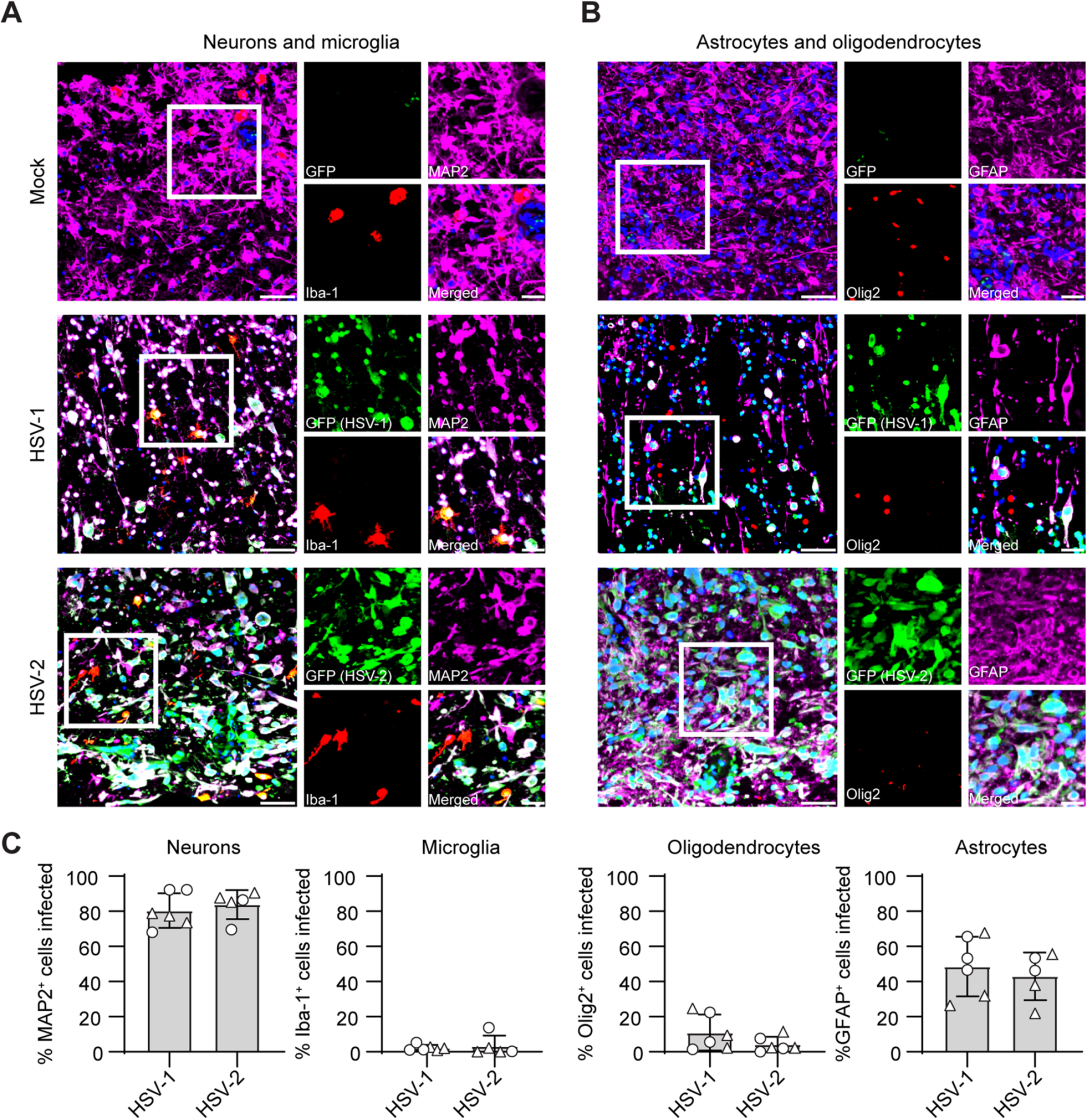
HSV-1 and HSV-2 preferentially infect astrocytes and neurons in human fetal organotypic brain slice cultures. **A**, **B** Brain slices were infected with 100 plaque forming units per mL of HSV-1-GFP or HSV-2-GFP. At 72 h post-infection, brain slices were fixed and longitudinally sectioned. Triple immunofluorescence staining of brain slice sections using antibodies against GFP (green color) expressed by both HSV-1 and HSV-2 strains combined with (**A**) MAP2 (neurons; magenta color) and Iba1 (microglia; red color) or (**B**) GFAP (astrocytes; magenta color) and Olig2 (oligodendrocytes; red color). All staining combinations have been counterstained with Hoechst (nuclear stain; blue color). Scale bar: 10 µm and 20 µm (inserts). **C** Quantification of the in situ data obtained at three separate levels of brain slices generated from two subjects (filled circles and triangles). Percentage marker-positive cells of all virus-infected cells are provided

To investigate the viral cell tropism, IF stainings were performed on hfOBSC sections to identify and quantify the cell types infected. At 72 hpi, HSV-1 and HSV-2 mainly infected MAP2^+^ neurons and GFAP^+^ astrocytes, while only minor proportions (< 10%) of Iba1^+^ microglia and Olig2^+^ oligodendrocytes were infected (Fig. 3A, B). Analogous to human HSE brain tissues, no clear differences in cell tropism were observed between HSV-1 and HSV-2 [36–40]. In conclusion, these results indicated that hfOBSC were permissive to productive HSV-1 and HSV-2 infection and spread throughout the tissue.

### Inflammatory response upon HSV-1 and HSV-2 infection in hfOBSC

**Fig. 4:**
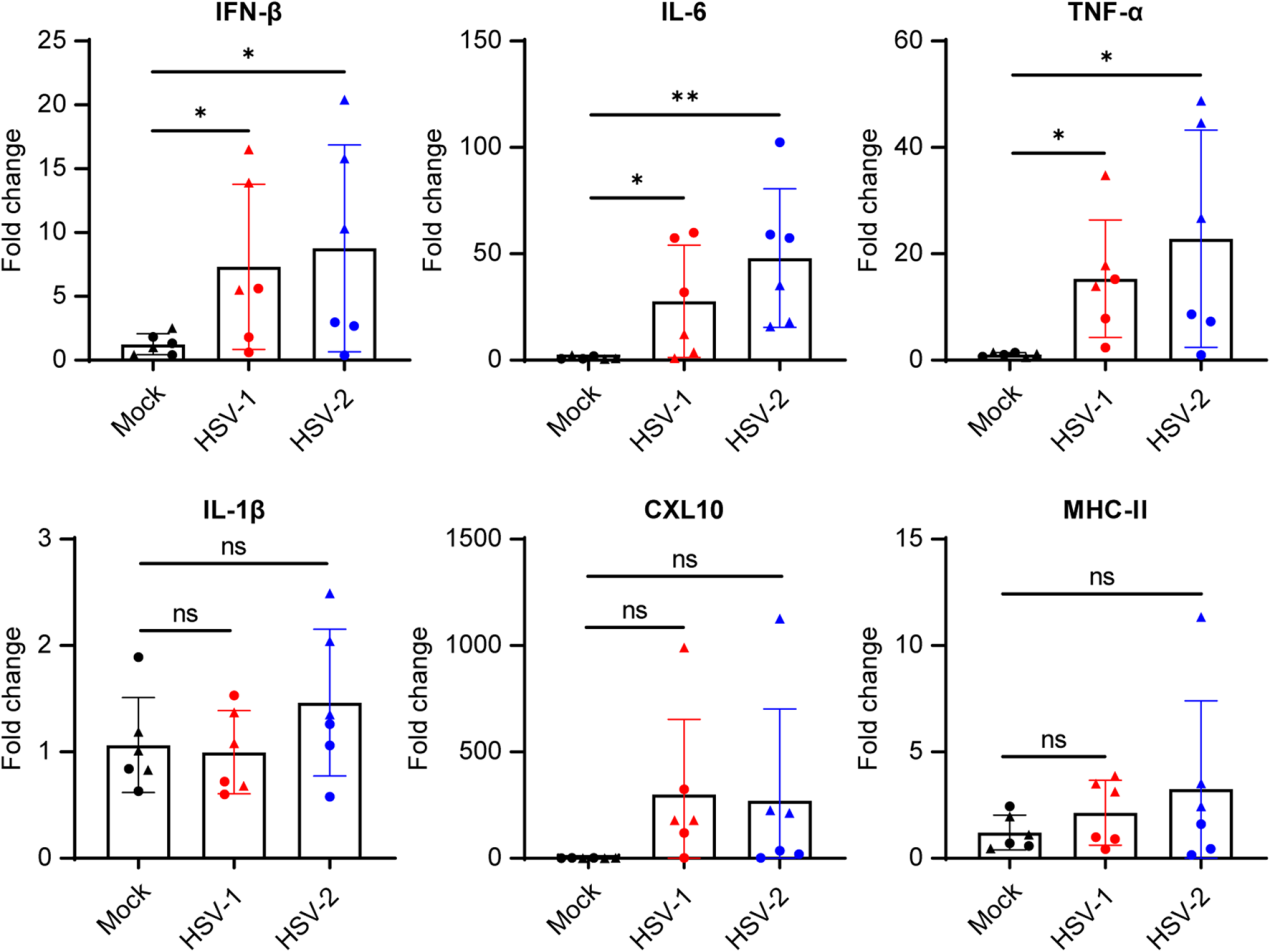
HSV-1 and HSV-2 infection induces a pro-inflammatory response in human fetal organotypic brain slice cultures. Brain slice cultures were infected with 10^6^ plaque forming units per mL of HSV-1-GFP or HSV-2-GFP. At 24 h post-infection, cellular RNA was isolated from HSV- and mock-infected fetal brain slices to determine transcript levels of the pro-inflammatory cytokines [i.e. *IL1B* (interleukin-1 β, IL-1β), *IFNB1* (IFN-β), *TNFA* (tumor necrosis factor alpha, TNF-α), *IL6* (interleukin-6, IL-6)], the chemokine *CXCL10* and *HLA-DRA* as a prototypic microglia activation marker (MHC-II*) by* RT-qPCR). Fold changes in gene expression, normalized to *GAPDH* and mock-infected brain slices using the 2^−ΔΔCt^ method, are shown. The data represent mean ± SD from two independent subjects (triangle and circle symbols) with three biological replicates each. Statistical significance was determined using Two-tailed Student’s *t* test. *p < 0.05, **p < 0.01, ***p < 0.001. ns, not significant

**Fig. 5:**
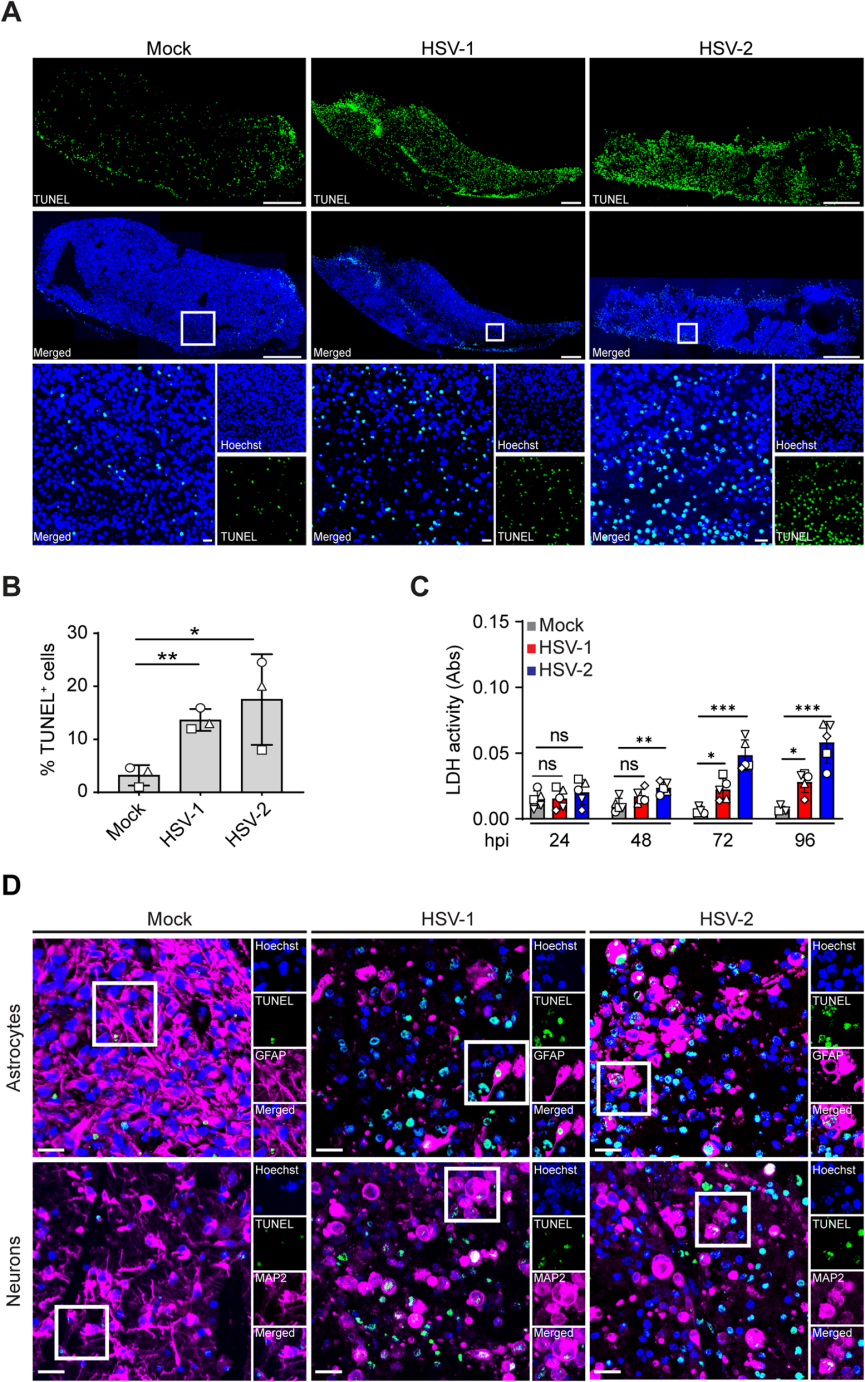
HSV-1 and HSV-2 infection induces cell death in human fetal organotypic brain slice cultures. **A** Immunofluorescence staining for terminal deoxynucleotidyl transferase dUTP nick end labeling (TUNEL; green color) counterstained with Hoechst (nuclear stain; blue color) on transverse sections cut from brain slices that were infected with GFP-expressing HSV-1 and HSV-2 (10^6^ plaque-forming units/mL) or sham-infected (mock), and subsequently cultured for 72 h post-infection (hpi). Upper 2 rows show stainings on the whole brain slice, with specific regions (boxed) enlarged and shown in the lower row. Scale bar: 20 µm. **B** Quantification of the percentages of TUNEL-positive cells in mock-, HSV-1- and HSV-2-infected brain slices shown in panel A. Data are mean percentage TUNEL-positive cells ± SD of three independent subjects. **C** Data of lactate dehydrogenase (LDH) assay on conditioned medium of mock, HSV-1, or HSV-2 infected brain slices shown in **A** and **B**. Data shown as the mean absorbance ± SD of 5 independent subjects. Two-tailed Student’s *t* test for **B** and **C**: *p* > 0.05 (ns); *p ≤ 0.05; **p ≤ 0.01; ***p ≤ 0.001. **D** Brain slices treated as in panel A were subjected to double immunofluorescence staining for TUNEL (green color) and GFAP (astrocytes; magenta color) or MAP2 (neurons; magenta color). Hoechst, (nuclear stain; blue) and scale bar = 20 µm. Images are representative of three independent subjects

Innate immune responses of CNS-resident cells, including neurons and especially microglia, prevent HSV spread within the brain [41, 42]. Indeed, genetic studies of patients with viral brain infections as well as experimental cell and animal models have provided evidence for the pivotal role of these immune mechanisms, which mediate a first line of antiviral control without significantly triggering inflammatory activities [41, 42]. To characterize the neuro-inflammatory response induced by HSV infection in the hfOBSC model, we determined the expression of selected proinflammatory cytokines and chemokines (IFN-β, IL-1β, TNF-α, IL-6 and CXCL10), previously described to be key factors in neuroinflammation and HSV infection [43–46], and the microglial activation marker HLA-DR [25]. Hereto, we performed RT-qPCR on RNA extracted from mock-infected hfOBSC or hfOBSC infected with 10^6^ PFU/mL HSV for 24 h. We observed a significant increase of *IFNB1, IL6* and *TNFA*, but not *IL1B*, *CXCL10* and *MHC-II* mRNA expression in response to both HSV-1 and HSV-2 infection (Fig. 4). The absence of HSV-induced MHC-II upregulation on microglia—despite the observed pro-inflammatory profile—was confirmed by IF staining on HSV-infected hfOBSC sections (Additional file 3: Fig. S3). The ability to activate microglia by IFN-γ stimulation of cultured hfOBSC, demonstrated by induction of MHC-II expression (Additional file 3: Fig. S3), indicated that the inability of a productive HSV infection to activate microglia is most likely not due to microglial dysfunctionality to respond to activation stimuli [25]. Furthermore, we observed an inflammatory phenotype in the astrocytes, as evidenced by morphological changes characterized by a decrease in astrocytic branching in response to the HSV-1 and HSV-2 in the infected hfOBSC (Fig. 5D). Overall, HSV-1 and HSV-2 infections in the hfOBSC induced an intensive upregulation of pro-inflammatory genes, shedding light on the innate immune response triggered with these viruses within the hfOBSC model.

### HSV-1 and HSV-2 infection induces cell death in astrocytes and neurons

HSE is associated with profound inflammation and extensive death of neurons and astrocytes, as well as lower numbers of oligodendrocytes and microglia in HSE [8, 47, 48]. Cell death in particular is thought to provide a significant contribution to virus-induced encephalitis and HSE pathogenesis [43, 49, 50]. To assess the extent of cell death during HSV infection in vivo as well as in the hfOBSC platform, TUNEL assays were performed on tissue sections from HSE patients and hfOBSC sections (Fig. 5A). The TUNEL assay facilitates the histological identification of dead cells by staining for fragmented DNA [51]. First, brain sections of two human HSV-1 and HSV-2 encephalitis cases were analyzed, which showed high numbers of TUNEL-positive cells in HSE lesions (Additional file 2: Fig. S2A). Next, the induction of cell death was investigated in mock- and HSV-infected hfOBSC. Inoculation of human brain slices with 10^6^ PFU/mL HSV-1-GFP or HSV-2-GFP led to a significant increase in the amount of TUNEL^+^ cells relative to the mock control at 72 hpi (Fig. 5B). In line with these results, LDH release steadily increased between 24 and 96 hpi following HSV-1 or HSV-2 infection, whereas it remained low in the mock-infected hfOBSC (Fig. 5C).

To determine which cell types were undergoing cell death the TUNEL assay was combined with IF staining for the four major CNS cell types. In both HSV-1- and HSV-2-infected hfOBSC, the majority of TUNEL^+^ cells were either GFAP^+^ astrocytes or MAP2^+^ neurons. These data resembled the preferential cell death of astrocytes and neurons in brain tissues of both HSE patients (Additional file 2: Fig. S2B). In conclusion, the data demonstrate that both HSV-1 and HSV-2 infection induced high levels of cell death in the hfOBSC model, predominantly in neurons and astrocytes, which is in line with in situ analysis of human HSE brain specimens reported here and by others previously [36, 37, 43, 52].

### HSV-1 and HSV-2 induce necroptosis in hfOBSC

**Fig. 6:**
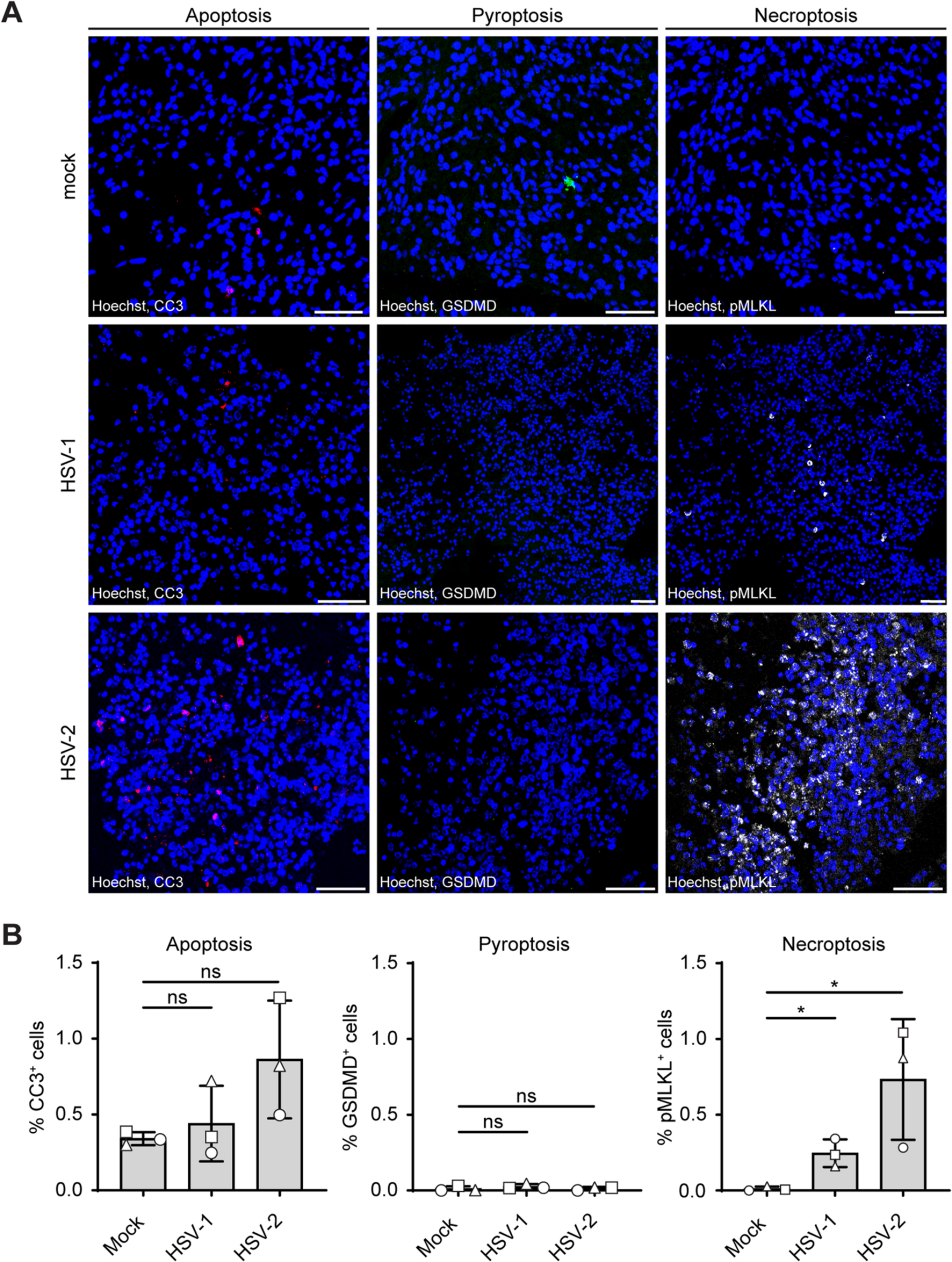
HSV-1 and HSV-2 infection induces necroptosis in human fetal organotypic brain slice cultures. **A** In situ immunofluorescence analysis of programmed cell death in HSV- and mock-infected human brain slices cultured until 72 h post-infection. Consecutive brain slice sections were stained for the apoptosis marker cleaved caspase 3 (CC3; red color), the pyroptosis marker gasdermin D (GSDMD; green color), or the necroptosis marker phosphorylated MLKL (pMLKL; white color). Images are representative of three subjects. Hoechst, nuclear stain (blue) and scalebar = 50 µm. **B** Quantification of the stainings obtained for **A**. Data are shown as mean percentage of marker-positive cells ± SD of three subjects. Two-tailed Student’s *t* test: *p* > 0.05 (ns) and *p* ≤ 0.05 (*)

Besides the direct cytopathic effect of HSV on brain cells, the virus may also induce cell damage indirectly by programmed cell death (PCD) [53]. PCD is a tightly regulated process that plays pivotal roles in tissue homeostasis as well as antiviral defense to control infection [54]. Various PCD pathways can be induced by HSV, which include non-inflammatory apoptosis and autophagy and inflammation inducing PCD including pyroptosis and necroptosis [37, 50, 53, 54]. Since we observed robust induction of cell death in HSV-infected hfOBSC, we determined whether this involved specific PCD pathways. We inoculated hfOBSC with 10^6^ PFU/mL HSV-1-GFP and HSV-2-GFP and determined three HSV-induced PCD pathways (i.e. apoptosis, pyroptosis and necroptosis) by in situ analysis of brain slice sections at 3 dpi (Fig. 6A). Expression of the following markers were used to differentiate between the three PCD pathways: cleaved caspase 3 (CC3) for apoptosis, gasdermin D (GSDMD) for pyroptosis and phosphorylation of mixed lineage kinase domain-like protein (pMLKL) as a marker for necroptosis [43, 53]. Whereas neither HSV subtype induced expression of GSMD, HSV-2 infection showed a trend to increased CC3 expression. Notably, productive infection with both HSV-1 and HSV-2 significantly induced expression of pMLKL (Fig. 6B). These results demonstrate that HSV-2 infection could lead to increased levels of apoptosis, while both viruses induced necroptotic cell death in hfOBSC.

## Discussion

The highly prevalent HSV-1 and HSV-2 are the most common causes of infectious encephalitis worldwide, with a disparate burden in individuals older than 50 or less than 1-year old [55, 56]. The cellular and molecular events that occur within the HSV-infected brain that lead to HSE are largely unknown.

Understanding the pathogenesis of HSE and other neurotropic virus infections requires the development of accurate and relevant model systems. Traditional two-dimensional (2D) cell cultures fail to capture the complex cellular interactions and three-dimensional architecture of human brain tissue, limiting their ability to reproduce the intricacies of brain development and diseases like HSE [57, 58]. Animal models, while valuable in elucidating HSE pathogenesis, exhibit variations in immune responses, cellular composition, and developmental stages among different species [58, 59]. As a result, translating results from animal models to the human context, especially for human-specific pathogens like HSV, has proven challenging [60, 61]. More sophisticated cell culture model systems such as brain organoids, although they exhibit self-organization and partial recapitulation of specific brain structures, fall short in replicating the precise cellular composition, cellular diversity, and complex structural organization observed in in vivo human brain tissue [62, 63]. To address these limitations, we have developed the hfOBSC model system presented here. During the gestational period of 17–20 weeks, human fetal brain development undergoes a dynamic process, giving rise to a diverse array of cell types, including neural progenitors, radial glia, intermediate progenitors and migrating neurons [64, 65]. This developmental stage culminates in the presence of mature cells such as neurons, microglia, astrocytes and oligodendrocytes that form a complex 3D cellular landscape. In this study, we showed that the hfOBSC model offers a promising alternative with preserved three-dimensional architecture and includes all four major human CNS cell types (i.e., neurons, microglia, astrocytes and oligodendrocytes). This novel human organotypic brain culture model enabled us to investigate the interplay between virus infection and PCD, showing that both HSV-1 and HSV-2 induce a pro-inflammatory response and necroptosis in human brain tissue, offering leads that can be explored for targeted therapeutic interventions in HSE and potentially other virus-induced brain diseases.

We have shown data demonstrating that the hfOBSC model resembles the cell tropism and neuropathology of HSV-1 and HSV-2 that is observed in affected brain tissue of human HSE cases [36, 37, 43, 52]. Interestingly, we consistently observed that the pattern of HSV-1 and HSV-2 infection and spread, as measured by GFP reporter fluorescence, appeared to be different (Fig. 2). While it is possible that this reflects a difference in neurovirulence between HSV-1 and HSV-2, comparable levels of viral DNA were released in supernatant of infected hfOBSC suggesting that both viruses replicated similarly. Therefore, a more likely explanation is the difference in reporter gene expression used for both viruses. Whereas for HSV-1 GFP is expressed as an N-terminal tagged fusion to the viral protein VP16 under control of the cognate HSV-1 VP16 promotor [23], GFP is ectopically expressed by a CMV promoter in HSV-2 [24]. As a result, the cellular localization and abundance, and hence fluorescence pattern could be different between both viruses.

Notably, our observations in HSV-infected brain slices revealed a pronounced occurrence of reactive astrocytes, characterized by morphological changes such as decreased branching. This transformative process actively contributes to an inflammatory phenotype, ultimately leading to cell death [49, 66–68]. In addition, our investigation into the inflammatory immune response revealed a complex interplay between antiviral and pro-inflammatory signals, as evidenced by upregulation of cytokines IFN-β, TNF-α and IL-6. These findings underscore the relevance and translational value of our hfOBSC model in studying HSE pathogenesis. Moreover, the utilization of human fetal tissue in this system establishes a valuable platform to study the neurovirulence of other TORCH pathogens as well as emerging neurotropic viruses that pose an increasing risk to human health worldwide [1, 69].

In previous studies, apoptosis was identified as the primary mode of PCD observed across different HSE models [36, 43, 70–72]. Apoptosis involves controlled cellular dismantling, and its activation is often associated with the host's attempt to limit viral spread and inflammation [73]. While these studies have shed light on the apoptotic pathways triggered during HSE, the involvement of necroptosis, a regulated form of necrosis controlled by specific signaling pathways [37, 50, 53, 54], is largely unknown. In contrast to previous studies on human (neonatal) HSE cases, which also reported apoptotic cell death as major neuropathological feature [43, 52, 70], our hfOBSC model shows only a trend toward HSV-induced apoptosis alongside a significant induction of HSV-induced necroptosis. These observations, coupled with the upregulation of specific cytokines in our hfOBSC, align with a previous study that demonstrated the amplification of TNFα production by type I interferon leading to necroptotic cell death [74], which explains the observation that both HSV-infected and surrounding uninfected cells are undergoing cell death in the brain of HSE patients [43, 74]. Notably, recent studies have highlighted the pivotal role of the cytoplasmic protein RIPK3, a key player in necroptosis, in modulating the severity of HSE [75, 76]. Future studies are warranted to detail the different PCD pathways in HSE during the development of disease upon intracerebral HSV infection.

The hfOBSC system provides a valuable platform to study the early innate immune response to HSV infection of CNS-resident cells. A limitation of our study is the apparent inability to obtain information on the later stages leading to HSE that involve the pivotal role of infiltrating immune cells including macrophages and T-cells into the infected brain [7, 10–12]. However, it is possible to partially mimic this in future studies using co-cultures of hfOBSC and spleen-derived leukocytes from the same donor. A second limitation is that the brain specimens were obtained following second-trimester surgical abortions, at which stage it is difficult to determine the anatomical orientation of the brain tissue fragments. Although measures were taken to ensure that the fetuses used for tissue collection did not exhibit major anatomical deformities or trisomy, the possibility of rare genetic alterations cannot be ruled out. Notably, we did not observe extensive variation between our biological donor replicates, which emphasizes the reproducibility of our findings. Therefore, the wider use of hfOBSC in future virological and non-virological studies will be important to fully appreciate donor variability and demonstrate robustness of our hfOBSC model.

In conclusion, we developed a novel human fetal organotypic brain slice model in which the viability of the major CNS resident cells, including microglia, and the tissue architecture is maintained for at least 14 days in culture. The cell tropism and neurovirulence of HSV-1 and HSV-2 in the hfOBSC model resembled observations in affected brain tissue of HSE cases [36, 37, 43, 52]. These data emphasize its potential to study aspects of pathogenesis of HSV and other neurotropic viruses in human brain tissue in vitro. While further research is needed to completely characterize the intricacies of the model, we believe that the hfOBSC model holds promise as a potential preclinical tool to explore the efficacy and safety of novel therapeutic intervention strategies aimed at targeting viral replication and detrimental host responses in human brain tissue.

### Supplementary Information

**Additional file 1: Figure S1. **Generation and metabolic activity of cultured human fetal organotypic brain slices. (**A**) Representative images showing unprocessed human fetal brain tissue and (**B**) selection of cortical human fetal brain tissue, as indicated by the white arrow, for subsequent slicing. (**C**) Agarose pieces were glued to the vibratome platform, as indicated by the white arrows. (**D**) Vertical and (**E**) horizontal orientation of the brain slice tissue dimension were employed to ensure standardization of the brain slice size. (**F**) Collection of the human fetal brain slice tissue in six separate collection chambers in artificial cerebrospinal fluid (aCSF) under constant oxygenation (95% O_2_, 5% CO_2_), as indicated by the white arrow. (**G**) Slicing of fetal brain slices was performed in aCSF under constant oxygenation using a vibratome. The slices were kept in an icocold environment to decrease cellular metabolic activity. (**H**) Viability assessment of a human fetal organotypic brain slices showing metabolic activity (purple) over a 14-days culture period using the 3-(4,5-dimethylthiazol-2-yl)-2,5-diphenyltetrazolium bromide (MTT) assay. Images are representative of two independent subjects.

**Additional file 2: Figure S2. **Neurons and astrocytes are the primary cell types undergoing cell death in brains of HSV-1 and HSV-2 encephalitis patients. (**A**) Immunofluorescence staining for terminal deoxynucleotidyl transferase dUTP nick end labeling (TUNEL; green color) on brain tissues sections of neonatal HSV-1 and HSV-2 encephalitis (HSE) cases. Hoechst (nuclear stain; blue color) and scalebar = 50 µm. (**B**) Brain tissue sections of HSV cases shown in panel A were subjected to double immunofluorescence staining for TUNEL (green color) in combination with GFAP (astrocytes; magenta color) or MAP2 (neurons; magenta color). Hoechst (nuclear stain; blue) and scale bar = 50 µm.

**Additional file 3: Figure S3.** HSV-1 and HSV-2 infection does not lead to upregulation of MHC-II expression in microglia. Brain slices were infected with 10^6^ plaque forming units per mL of HSV-1-GFP or HSV-2-GFP, or treated with 1,000 U/mL recombinant human IFN-γ. At 24 h post-infection, brain slices were fixed and longitudinally sectioned. Triple immunofluorescence staining of brain slice sections was performed using antibodies against GFP (green color) combined with Iba1 (microglia; red color) and MHC-II (prototypic microglia activation marker, white color). All staining combinations were counterstained with Hoechst 33,342 (nuclear stain; blue color). Scale bar: 50 µm

## Data Availability

The datasets and scripts used and/or analyzed during the current study are available from the corresponding author upon reasonable request.

## Abbreviations

aCSF: Artificial cerebrospinal fluid
CC3: Cleaved caspase 3
CNS: Central nervous system
FFPE: Formalin-fixed paraffin embedded
GFAP: Glial fibrillary acidic protein
GFP: Green fluorescent protein
GSDMD: Gasdermin D
hpi: Hours post-infection
HSE: Herpes simplex encephalitis
HSV-1: Herpes simplex virus type 1
hfOBSC: Human fetal organotypic brain slice culture
IF: Immunofluorescence
IHC: Immunohistochemistry
LDH: Lactate dehydrogenase
MAP2: Microtubule-associated protein 2
MTT: 3-(4,5-Dimethylthiazol-2-yl)-2,5-diphenyltetrazolium bromide
Olig2: Oligodendrocyte transcription factor 2
PCD: Programmed cell death
PFU: Plaque-forming units
pMLKL: Phosphorylation of mixed lineage kinase domain-like protein
RT-qPCR: Real-time quantitative PCR
TUNEL: Terminal deoxynucleotidyl transferase mediated dUTP nick end labeling
