# Supplementary figures and images for: Herpes simplex virus infection induces necroptosis of neurons and astrocytes in human fetal organotypic brain slice cultures
Source: J Neuroinflammation. 2024 Feb 1;21:38. doi: 10.1186/s12974-024-03027-5 (PMC10832279; doi:10.1186/s12974-024-03027-5)

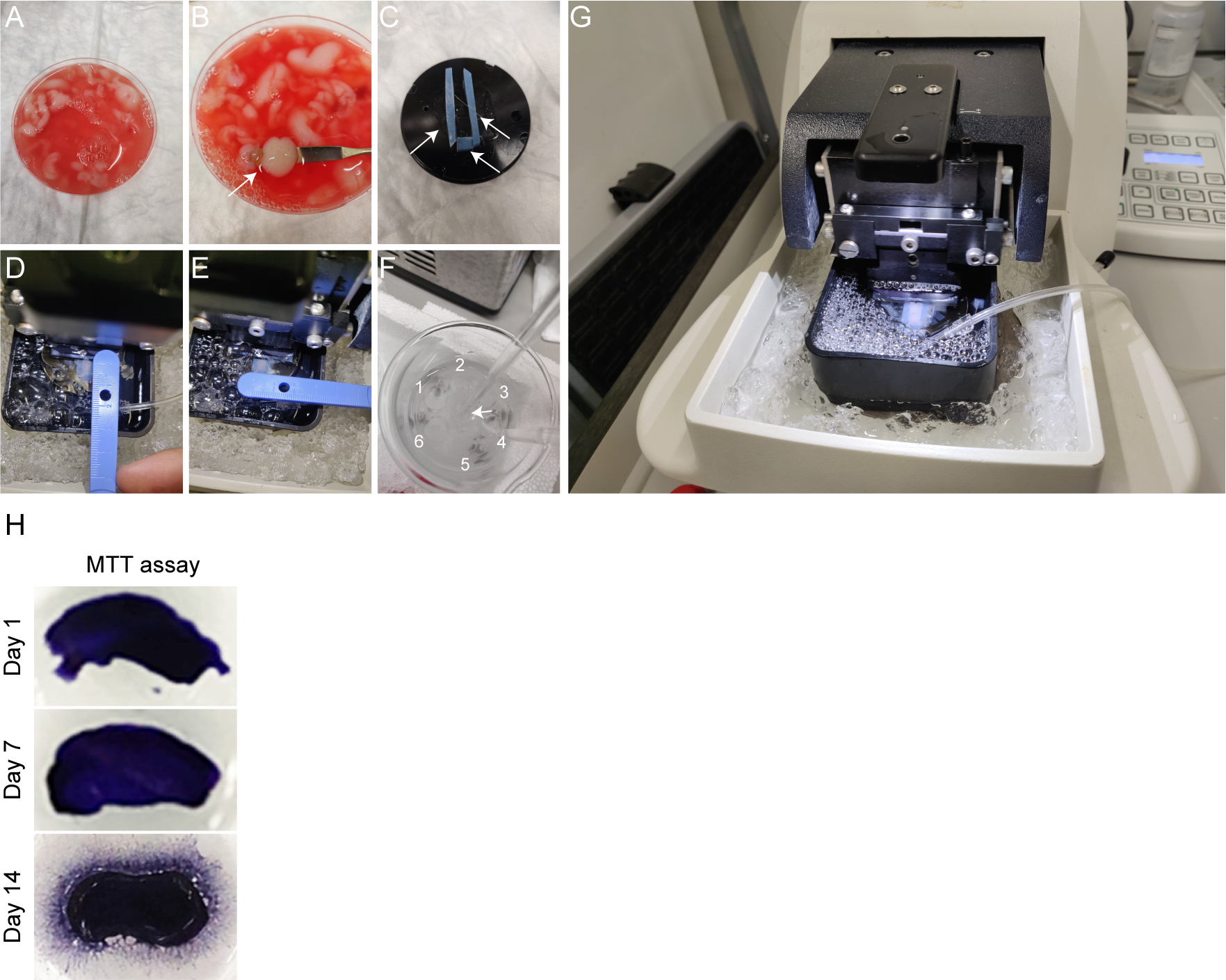

Supplement: Supplementary file 1 — Additional file 1: Figure S1. Generation and metabolic activity of cultured human fetal organotypic brain slices. (A) Representative images showing unprocessed human fetal brain tissue and (B) selection of cortical human fetal brain tissue, as indicated by the white arrow, for subsequent slicing. (C) Agarose pieces were glued to the vibratome platform, as indicated by the white arrows. (D) Vertical and (E) horizontal orientation of the brain slice tissue dimension were employed to ensure standardization of the brain slice size. (F) Collection of the human fetal brain slice tissue in six separate collection chambers in artificial cerebrospinal fluid (aCSF) under constant oxygenation (95% O2, 5% CO2), as indicated by the white arrow. (G) Slicing of fetal brain slices was performed in aCSF under constant oxygenation using a vibratome. The slices were kept in an icocold environment to decrease cellular metabolic activity. (H) Viability assessment of a human fetal organotypic brain slices showing metabolic activity (purple) over a 14-days culture period using the 3-(4,5-dimethylthiazol-2-yl)-2,5-diphenyltetrazolium bromide (MTT) assay. Images are representative of two independent subjects. [file 12974_2024_3027_MOESM1_ESM.tif]

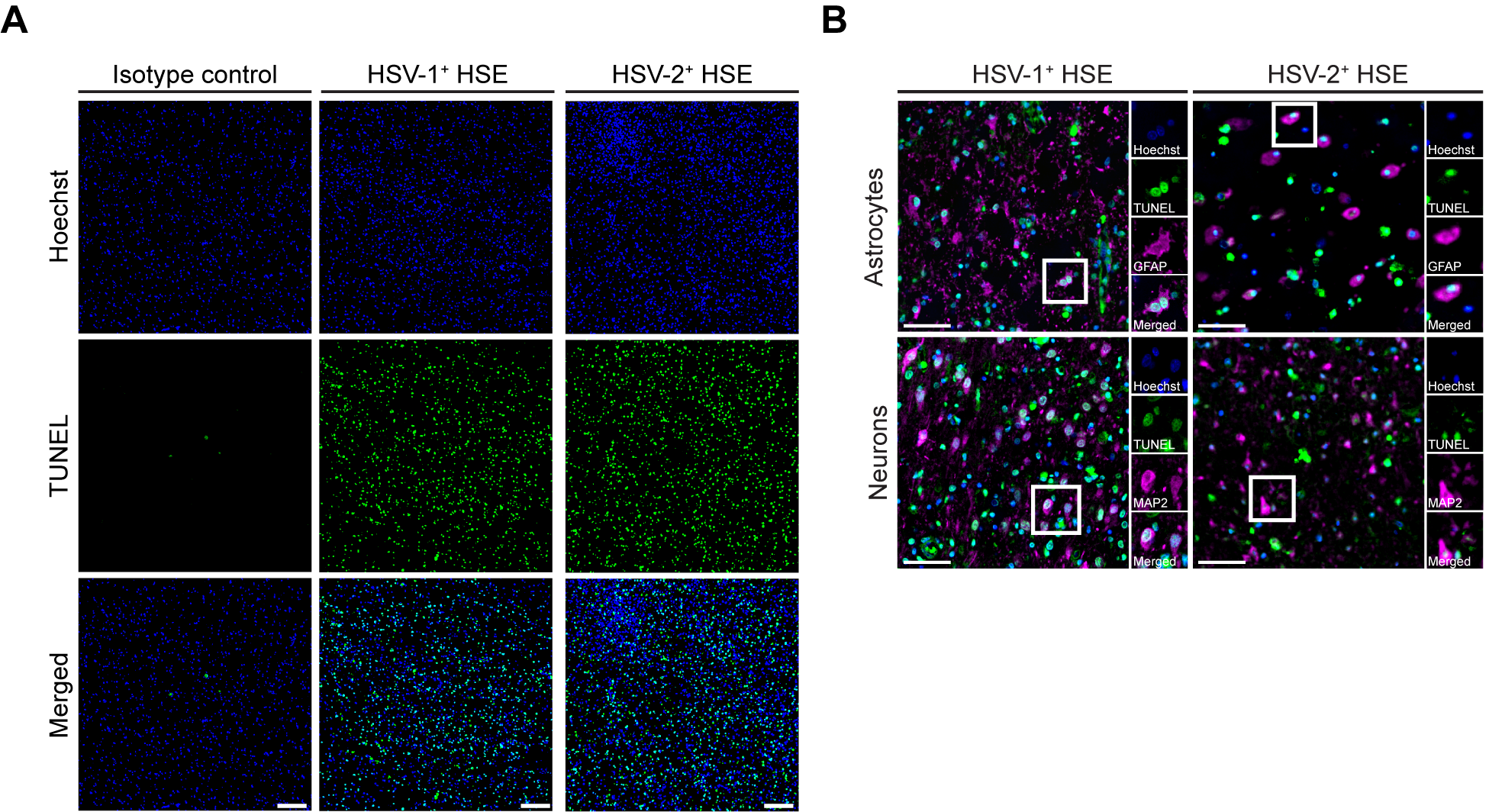

Supplement: Supplementary file 2 — Additional file 2: Figure S2. Neurons and astrocytes are the primary cell types undergoing cell death in brains of HSV-1 and HSV-2 encephalitis patients. (A) Immunofluorescence staining for terminal deoxynucleotidyl transferase dUTP nick end labeling (TUNEL; green color) on brain tissues sections of neonatal HSV-1 and HSV-2 encephalitis (HSE) cases. Hoechst (nuclear stain; blue color) and scalebar = 50 µm. (B) Brain tissue sections of HSV cases shown in panel A were subjected to double immunofluorescence staining for TUNEL (green color) in combination with GFAP (astrocytes; magenta color) or MAP2 (neurons; magenta color). Hoechst (nuclear stain; blue) and scale bar = 50 µm. [file 12974_2024_3027_MOESM2_ESM.tif]

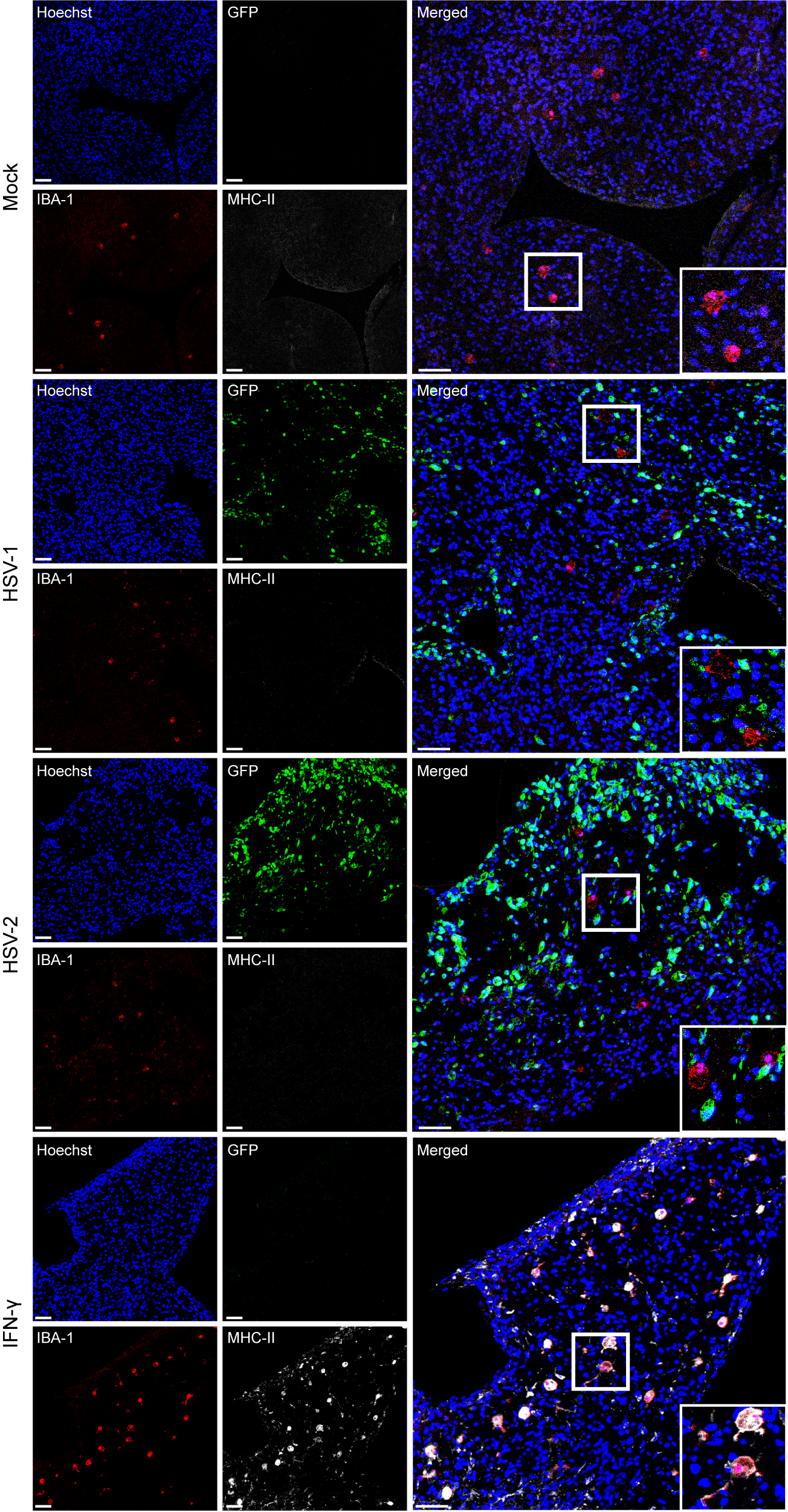

Supplement: Supplementary file 3 — Additional file 3: Figure S3. HSV-1 and HSV-2 infection does not lead to upregulation of MHC-II expression in microglia. Brain slices were infected with 106 plaque forming units per mL of HSV-1-GFP or HSV-2-GFP, or treated with 1,000 U/mL recombinant human IFN-γ. At 24 h post-infection, brain slices were fixed and longitudinally sectioned. Triple immunofluorescence staining of brain slice sections was performed using antibodies against GFP (green color) combined with Iba1 (microglia; red color) and MHC-II (prototypic microglia activation marker, white color). All staining combinations were counterstained with Hoechst 33,342 (nuclear stain; blue color). Scale bar: 50 µm [file 12974_2024_3027_MOESM3_ESM.tif]
